# Patient-centered outcomes used in pediatric focused manual therapies research studies: a secondary data analysis of a systematic review

**DOI:** 10.1186/s41687-021-00305-1

**Published:** 2021-04-01

**Authors:** Beth Carleo, Kristian Anderson, Carol Parnell Prevost, Katherine A. Pohlman

**Affiliations:** 1 Palmer College of Chiropractic, 4777 City Center Parkway, Port Orange, FL 32129 USA; 2 Performance Chiropractic, 4350 South Washington Street Suite 100, Grand Forks, ND 58201 USA; 3 Parker University, 2540 Walnut Hill Lane, Dallas, TX 75229 USA

## Abstract

**Background:**

Patient-reported outcome measurements (PROM) are instruments that seek a patient’s health or functional status. Inclusion of standardized PROMs in research studies and clinical practice provides a more comprehensive understanding of an intervention from the patient’s viewpoint. This secondary analysis identified PROM usage and appropriateness of references for property measurements from clinical trials included in a recent systematic review of pediatric manual therapy.

**Methods:**

All included manuscripts within a recent systematic review had two authors extract PROM and associated property measurement data, including the property measurements supporting citations. Inclusion criteria for the articles were published clinical trials (observational or experimental) of pediatric children receiving manual therapy (any profession) for any condition between 2001-March 2018. For each PROM’s associated property measurement, two authors used the COSMIN study design checklist to appraise the quality of the cited study to evaluate the property measurement*.*

**Results:**

Of the 50 manuscripts included in the systematic review, 20 manuscripts reported the use of 52 PROMs. Of the 52 PROMs assessed, 31 did not make a statement about the instrument’s property measurement, 7 PROMs had property measurements stated but not referenced, 4 PROMs stated that the property measurement information was unknown, and 10 had property measurement data with reference(s). These 10 PROMs with referenced property measurements were from 7 unique PROMs: constipation assessment scale, satisfaction visual analog scale (VAS), crying time diary, sleep diary, fear avoidance belief questionnaire (FABQ), pain VAS, and autism treatment evaluation checklist. The assessment of the referenced property measurements found that several property measurement’s dimensions had not been assessed and those that had were evaluated were done so with poor or fair standards.

**Conclusions:**

This secondary analysis finds that clinical studies of pediatric manual therapy lack consistent use of PROMs with high quality property measurements. Further research to establish and implement PROMs to be used in future research studies and in clinical settings should become a priority for professions using manual therapy in children.

**Supplementary Information:**

The online version contains supplementary material available at 10.1186/s41687-021-00305-1.

## Introduction

In recent years, researchers have produced multiple literature reviews on manual therapy use within the pediatric population [1]. Pediatric manual therapy can include spinal manipulative therapy, mobilization, chiropractic/osteopathic manipulative therapy, or cranial-sacral therapy [1]. While differences exist between them, all reviews conclude a paucity of evidence for effectiveness, mostly due to methodological flaws within the individual included studies. A consistent methodological flaw was the lack of standardized patient-reported outcome measurements (PROMs) [1].

PROMs are instruments or tools that seek a patient’s response to their health, quality of life, or functional status from their health care or treatment [2]. They allow health outcomes to be measured throughout a treatment plan directly from the patient’s perspective. Research of chiropractic, a profession that commonly uses manual therapy, frequently uses PROMS focused on musculoskeletal issues in the adult population [3].

There are many obstacles to the adoption of PROMs in clinical practice. Many clinicians lack understanding about the instruments and have concerns that the time it will take to administer PROMs would increase their workload [4]. Additional challenges include achieving higher patient participation rates, especially among older, sicker, and illiterate patients [5]. Despite the understanding of PROM’s importance in clinical practice, the routine use of these tools is limited [6].

This secondary data analysis aimed to identify PROMs reported in published clinical trials (observational or experimental) of children receiving manual therapy (any profession) for any condition between 2001-March 2018. For the identified PROMs, two authors extracted property measurement information and evaluated current CONSORT guidelines for reporting property measures [7]. When citations for property measurements were referenced, authors further used COSMIN checklists to evaluate and score the quality of the methods that were conducted to assess the stated property measurement [8, 9].

## Methods

### Initial systematic review

Details of the initial systematic review used to identify studies for this research project have been previously published [1]. In brief, the systematic review’s inclusion criteria were: full-text reports of randomized controlled trials (excluding feasibility studies without outcome measures) or observational studies with pre and post measurements (case reports were excluded) that included two or more children under the age of 18 treated with any form of manual therapy from any healthcare provider and published in English. This research project was registered on PROSPERO: CRD42016033681 and the initial systematic review [1] was also registered there: CRD42018091835.

### Patient-reported outcome measures (PROMs)

Every included study in the original SR was reviewed by one of the current authors and validated by another author. PROMs used in a study and details stated regarding the PROM’s property measurements were extracted. The extracted data were categorized into CONSORT reporting guidelines [7]. Those that did follow CONSORT guidelines were categorized as: “Stated that property measurements were unknown” or “Stated with references”. Those that did not follow CONSORT guidelines were categorized as: “None stated,” or “Stated, but not referenced”. If a reference was cited but then discovered by the authors not to include property measurement evaluations, these manuscripts were changed to “Stated, but not referenced.” All information was tabulated and reported.

### PROM property measurement assessment

For unique PROM with property measurements referenced, two authors further evaluated the referenced manuscript. If the same PROM was cited by more than one manuscript, all citations from any of the manuscripts were evaluated for the PROM. First, the referenced manuscript was assessed for which property measurement dimension was being evaluated using the definitions determined by the COSMIN guidelines [8]. Exact property measurement dimensions are: Reliability, Internal Consistency, Measurement Error, Content Validity (including face validity), Criterion Validity, Construct Validity (including Structural Validity, Hypothesis-testing Validity, Cross-cultural Validity), Responsiveness, and Interpretability.

The lead author assessed the property measurement of each article using the respective COSMIN study design quality checklist. The senior author independently evaluated assessments and consensus was reached by discussions between the two authors. Scores for each checklist item were based on a 4-point scale: ‘poor’, ‘fair’, ‘good’, or ‘excellent’ [9]. Based on the COSMIN recommendation, the overall quality score was determined by taking the lowest rating of any item in the checklist (‘worst score counts’).

## Results

### Patient-reported outcome measures (PROMs)

From the 50 studies that met the Parnell-Prevost [1] review’s inclusion criteria, 20 manuscripts (40%) reported the use of 52 PROMs, with 30 manuscripts (60%) not using any PROMs. Descriptive details of the 52 PROMs can be found in Supplement #1.

As shown in Table 1, the majority of the 52 PROMs did not make a statement regarding the PROM’s property measurements (*n* = 31, 59.6%). Of the articles that did make a statement on the PROM’s property measurements, 7 manuscripts had statements of property measurements existing with no citations; in addition to these, two of the PROMs stated and referenced property measurements, but the references did not include property measurement assessments. A total of 7 PROMs (13.5%) were categorized as “Stated, but not referenced”. In addition, 4 PROMS (7.8%) described the property measurements as unknown. Only 10 of the 52 PROMs (19.2%) had references to prompt a property measurement evaluation. These 10 PROMs were from 7 of the 50 initial manuscripts (14.0%).

**Table 1:** PROMs used in clinical trials of manual therapy in children and the assessment of their reported property measurements. For more information on the PROMs, please see Supplement #1

PROM and Number of Manuscripts	System / Condition being studied
**PROM with property measurement stated with references (*n* = 10)**	
Constipation Assessment Scale (*n* = 1)	Constipation and Cerebral Palsy
Satisfaction VAS (*n* = 1)	Constipation and Cerebral Palsy
Crying time diary (*n* = 2)	Infantile Colic
Sleep diary (*n* = 1)	Infantile Colic
Fear avoidance back questionnaire (*n* = 1)	Mechanical Low Back Pain
Pain VAS (*n* = 3)	Mechanical Low Back Pain, Cuboid Syndrome
Autism Treatment Evaluation Checklist (*n* = 1)	Autism
**PROM with property measurement stated, but not referenced (*n* = 7)**	
Global rating of change (*n* = 1)	Mechanical Low Back Pain
Low back pain severity (*n* = 1)	Low Back Pain
Patient specific functional scale (*n* = 1)	Mechanical Low Back Pain
Autism Research Institute - secretin outcomes survey (*n* = 1)	Autism
Scoliosis Quality of Life Index (*n* = 1)	Scoliosis
General Health Question from Quality of Life index (*n* = 1)	Chronic-Tension Type Headaches
Pain Intensity (*n* = 1)	Chronic Tension Type Headaches
**PROM with property measurement stated as unknown (*n* = 4)**	
Improvement rating (*n* = 1)	Suboptimal Infant Breastfeeding
Mother’s report of exclusivity of breastfeeding (*n* = 1)	Suboptimal Infant Breastfeeding
5-point subjective rating scale (*n* = 1)	Mechanical Low Back Pain
Modified Oswestry disability index (*n* = 1)	Mechanical Low Back Pain
**PROM with property measurement not stated (*n* = 31)**	
Defecation frequency (*n* = 1)	Constipation and Cerebral Palsy
24 h Crying time diary (*n* = 1)	Infantile Colic
Crying time diary (*n* = 1)	Infantile Colic
Diary for wet night frequency (*n* = 1)	Nocturnal Enuresis
Doctor classification system based on parental report (*n* = 1)	Infantile Colic
Dysfunctional voiding symptoms (*n* = 1)	Pediatric Dysfunctional Voiding
Reported ability to latch and ability to breastfeed (*n* = 1)	Suboptimal Infant Breastfeeding
Roland-Morris Disability Questionnaire (*n* = 1)	Low Back Pain
Frequency of medication use (*n* = 1)	Low Back Pain
Headache diary (*n* = 1)	Headache
Headache frequency (*n* = 1)	Chronic tension-type headaches
Improvement rating (*n* = 1)	Low Back Pain
Numeric Pain Rating Scale (*n* = 1)	Mechanical Low Back Pain
Quality of Life (*n* = 1)	Mechanical Low Back Pain
Satisfaction rating (*n* = 1)	Low Back Pain
Asthma severity and improvement (*n* = 1)	Asthma
Behavior scores (*n* = 1)	Otitis Media
Parent-reported occurrence (*n* = 1)	Otitis Media
Quality of life questionnaire (*n* = 1)	Asthma
Carer/Parent quality of life questionnaire (*n* = 1)	Cerebral Palsy
Fit and sleep Diaries (*n* = 1)	Cerebral Palsy
Parent assessment of child global health and sleep (*n* = 1)	Cerebral Palsy
Parent reported changes (*n* = 1)	Cerebral Palsy
Pediatric pain profile (*n* = 1)	Cerebral Palsy
Quality of life using Child Health Questionnaire (*n* = 1)	Cerebral Palsy
VAS to rate spasticity (*n* = 1)	Cerebral Palsy
Functional rating index (*n* = 1)	Scoliosis
Global Perceived effect scale (*n* = 1)	Upper cervical dysfunction
Parent questionnaire (*n* = 1)	Upper cervical dysfunction
Vegetative parameters questionnaire (*n* = 1)	Postural asymmetry
Visual analog scale for pain (*n* = 1)	Upper cervical dysfunctional

### Property measurement assessment

The 10 PROMs identified 7 unique and 3 repeat instruments. As shown in Table 2, at least one property measurement dimension was found for each PROM. Property measurement dimensions not assessed in any of the PROMs include: Measurement Error, Hypothesis-Testing Validity, and Cross-Cultural Validity.

**Table 2:** Methodological quality assessment of property measurements from PROMs used in research studies of children receiving manual therapy

	Reliability	Internal Consistency	Content Validity	Criterion Validity	Structural Validity	Responsiveness
Constipation Assessment Scale (*n* = 1) [10]	POOR				POOR	
Satisfaction VAS (*n* = 1) [10]					POOR	
Crying time diary (*n* = 2) [11, 12]				POOR (*n* = 2)		
Sleep diary (*n* = 1) [11]				FAIR		
Fear Avoidance Back Questionnaire (*n* = 1) [13]		POOR	FAIR		POOR	
Pain VAS (*n* = 3) [13]	FAIR (*n* = 1) POOR (*n* = 1)		POOR (*n* = 1) GOOD (*n* = 1)		POOR (*n* = 1)	POOR (*n* = 1)
Autism Treatment Evaluation Checklist (*n* = 1) [14]		POOR				

LEGEND: *VAS* Visual Analog Scale.

For the 6 property measurement dimensions assessed with the references, Structural Validity was the most common dimension assessed; however, all 4 assessments receiving ‘poor’ methodological quality score. Responsiveness was the least referenced property measurement dimension with one PROM (Pain VAS- visual analog scale), and it also received a ‘poor’ methodological quality score. Among all 16 property measurements assessed, the most common score was ‘poor’ (*n* = 11). A few received a ‘fair’ score (*n* = 4), with only Content Validity for pain VAS receiving a ‘good’ methodological quality score. Checklist evaluations for each PROM and property measurement can be found in Supplement #2.

## Discussion

This secondary analysis study found a lack of high-quality standardized PROMs reported in clinical studies of manual therapies for children. From the 50 studies reviewed, 52 PROMs were found to with 7 unique PROMs having appropriately referenced property measurements. When the methodology for these 7 PROMs’ property measurements were evaluated, most were of ‘fair’ or ‘poor’ quality. These findings are similar to those found when the spectrum of outcome measures used in pediatric orthopedic publications were evaluated [15]. Of their 2251 reviewed studies, only 11.5% used a PROM, with few having appropriate validation. These reviews signify a need to encourage the use of the same scales across multiple trials, which could more efficiently impact treatment strategies for the pediatric population.

Of the 7 PROMs identified with evaluated property measurements, the Autism Treatment Evaluation Checklist [14], Constipation Assessment Scale, and Satisfaction Question with a visual analog scale (VAS) [10] were each used in 1 study only and their property measurements were all found to be developed with ‘poor’ quality [10]. In a 2015 systematic review of measurement outcomes for children with autism [16] and a 2018 scoping review of constipation [17], neither recommended the use of these tools or any other tool because of the lack of well-developed property measurements. The satisfaction question is commonly measured, especially in musculoskeletal studies; however, it is usually measured with non-standardized, locally-devised tools [18]. All of these outcomes emphasize the need for more standardization of PROMs and their use.

Of the remaining PROMs with evaluated property measurements, both crying time and sleeping duration were collected using a diary format and had Criterion Validity evaluated as either poor or fair, respectively. The diary format is desired as it should reduce recall bias by collecting data ‘in the field’. However, diary methods requires a patient or a proxy to self-monitor, with adherence to this protocol shown to be unreliable [19]. With technological advances and better methodological understanding, diaries are still considered viable ways to enrich PROM data, especially for quality of life measures [20].

Studies with evaluated property measurements that looked specifically at musculoskeletal conditions identified 2 PROMs. The Pain VAS was the only PROM to be used in 3 studies. This measure had several property measurements evaluated with a range of quality from ‘poor’ to ‘good’. A Fear Avoidance Belief Questionnaire (FABQ) was also used and found to assess several property measurements with ‘fair’ or ‘poor’ quality ratings [13]. A recent commentary by Michaleff et al. highlighted the clinician’s challenges to assess pain [21]. They also provide 8 different age-appropriate suggestions for measuring self-report of pain intensity, which included a VAS, along with other validated scales that use color or faces. We recommend the use of one of the 8 scales in the age-appropriate clinical situations.

Importantly, the effort placed on selecting appropriate PROMs needs to include usability in the clinic setting in addition to clinical research. Stinson et al. suggest ease of use and scoring are points in the process where clinical research and clinical practice settings may present different needs [22]. There has been effort dedicated to identifying scales useful to the pediatric population for both settings (e.g., PedIMMPACT, PROMIS). McGrath et al. recommends PedIMMACT for the core outcome domains for both acute and chronic/recurring pediatric pain conditions [23]. PROMIS domains allow for a measure to capture the larger impact of an intervention on a pediatric pain condition [24]. Both measures are limited in use to those who are 5 years of age and older, leaving a void for those younger than this age.

This secondary data analysis is limited to studies included in the initial systematic review. While this limitation allows a more detailed review of the specific content area, these parameters do not allow for a specific review to be developed for this content. The limitation of only reviewing the citations for property measurements included in the studies also limited the potential measurement properties of each PROM; thus, the reporting is certainly incomplete. Another known limitation for all pediatric healthcare and research is the use of proxy-report by parent/caregiver. In the literature, proxy-report of child health has been shown to be contradictory, with both over- and under-estimates reported [25]. Further research is needed to better understand the effect of proxy-reporting of pediatric PROMs.

## Conclusion

This secondary analysis documents the need to develop high-quality PROMs on manual therapy for pediatric populations. Without such PROMs, manual therapy research, as well as practitioners using this therapy, are at a loss for an approach to collect valuable patient data that could best assess patient progress. Further research to establish and implement PROMs to be used in future research studies and in clinical settings should become a priority for professions using manual therapy in children.

## Supplementary Information

**Additional file 1.**

**Additional file 2.**

## Data Availability

All data generated or analyzed during this study are included in this published article [and its supplementary information files].

## Abbreviations

CONSORT: Consolidated Standards of Reporting Trials
COSMIN: COnsensus-based Standards for the selection of health Measurement Instruments
PROMIS: Patient Reported Outcomes Measurement Information System
PROMs: Patient Reported Outcome Measures
VAS: Visual Analog Scale
