# Supplementary material for: Patient-centered outcomes used in pediatric focused manual therapies research studies: a secondary data analysis of a systematic review
Source: J Patient Rep Outcomes. 2021 Apr 1;5:31. doi: 10.1186/s41687-021-00305-1 (PMC8017068; doi:10.1186/s41687-021-00305-1)
Supplement: Supplementary file 1 — Additional file 1. [file 41687_2021_305_MOESM1_ESM.docx]

**Supplement #1**

**Musculoskeletal (12 articles)**

Twelve articles met the inclusion criteria for the Musculoskeletal section. Two headache articles, Four Low Back articles, and one cuboid syndrome article utilized PCOs. Two pulled elbow, one Temporomandibular dysfunction, one infant headache and one clubfoot article did not use PCOs.

**Headache**

Borusiak-Headache Diary (Patient age 7 to15 years of age)

In Borusiak’s Headache Article a Headache Diary was used. The Diary included headache number of days, duration, missed school lessons, use of analgesics, and intensity/severity of complaints. “*(1) percentage of days with headache; (2) total duration of headache; (3) percentage of days with missing school lessons due to headache; (4) percentage of days with necessity of analgesic medication; and (5) intensity of headache.”* Comparisons were made between the reporting periods of the initial documentation period and the 2-month period following intervention. Property measurements for this Diary are not mentioned in the article.(Borusiak et al., 2010)

Przekop- Headache frequency (Patient age 13 to18 years of age)

Headache frequency was based on the average number of headaches per month in the preceding 3 months. Property Measurements were not mentioned on this PCO.Assessment occurred at baseline, 3 months, and 6 months. (Przekop et al., 2016)

Przekop-Pain Intensity (Patient age 13 to18 years of age)

Pain intensity was used by asking the following question: “Please rate your pain by circling the one number that best describes your pain at its worst in the last 24 hours.” This question appears as Question #3 from the Brief Pain Inventory. Ratings are on a scale ranging from 0 (no pain) to 10 (pain as bad as you can imagine). (Cleeland, Przekop). Property measurements stated in the article that reference article studies this scale for cancer patients and finds it "having demonstrated both reliability and validity across cultures and languages". Assessment occurred at baseline, 3 months, and 6 months.(Przekop et al., 2016)

Przekop-General Health Question and Health Interference Question (Patient age 13 to 18 years of age)

Two questions were taken from the modified Short Form Health Survey (SF-12) to assess General Health. (Ware Jr. J.E. Kosinski., Dewey, 2002) Questions taken from the form include: “ #1, “In general, would you say your health is . ” excellent (5), very good (4), good (3), fair (2), poor (1), and #12, “During the past 4 weeks, how much of the time has your physical health or emotional problems interfered with your social activities?” Scale ranges from none of the time (5) to all of the time (1).” Assessment occurred at baseline, 3 months, and 6 months. (Przekop et al., 2016) (Ware Jr. J.E. Kosinski., Dewey, 2002)

*The Ware textbook (reference received via ILL) states the reliability of SF-12 scale for general health is .75 in a general US population (physical function .78, bodily pain .73, mental health .87, physical component summary .89, mental component summary .86). Based on the text, the sample size does not include patients under the age of 18 (Table 10.2, pg. 65); page 159 says "results from 277 studies, the great majority of which were published by other researchers between 1995-2001." "This literature, which focuses primarily on the two SF-12 summary measures, generally supports their validity and interpretation as physical and mental health measures, as hypothesized in the first SF-12 User's Manual (Ware, Kosinski, Keller et al, 1995.)"*(Ware Jr. J.E. Kosinski., Dewey, 2002)

**Low Back Pain**

Hayden 5-point scale (Patient age 4 to 18 years of age)

The 5-point subjective scale used in the Hayden article addressing pediatric Low back pain patients gauged the change in the patient’s subjective pain using a 5-point Likert scale. The parameters were scale, 1 “worse,” 2 “same,” 3 “improved,” 4 “much improved,” and 5 “resolved”. (Mireau) The article did mention that the validity and reliability was unknown. This PCO was collected at each follow-up visit.(J. A. Hayden et al., 2003)

Hayden VAS (Patient age 4 to 18 years of age)

For the VAS used in this article, patients were asked to self-report using a pediatric visual analog scale the severity of their pain on 10cm VAS, the capstones included no pain (smiling face) and frowning face (biggest hurt you have ever had). “This scale, which has previously been tested for validity and reliability in pediatric populations,((Varni et al., 1987) has demonstrated reliability in adolescents with LBP problems.”(Staes et al., 2000) This PCO was collected at each follow-up visit. (J. A. Hayden et al., 2003)

Selhorst PSFS (Patient age 13 to 17 years of age)

The Patient Specific Functional Scale (PSFS) was used as a primary outcome in the Selhorst article for mechanical LBP. The patients nominated 3 activities that they are currently unable to perform due to the back pain that they were experiencing at the time of evaluation. Each of the 3 activities was then scored from 0 (not able) to 10 (able at preinjury level). “The PSFS has been shown to be more responsive than other low back outcome measures in adolescents and individuals with minimal activity limitations.” (A. M. Hall et al., 2011)(Fritz et al., 2005) The Scale was measured at baseline evaluation, 1 week, 4 weeks and 6 months. In this study the Physical therapy intervention was performed for 4 weeks.(Selhorst & Selhorst, 2015)

Selhorst NPRS (Patient age 13 to 17 years of age)

The Numeric pain-rating scale (NPRS) was used as the second primary outcome in the Selhorst study. The 11-point scale rated pain from 0 (no pain) to 10 (worst imaginable pain). This scale was used for current pain, best pain and worst pain in the past 24 hours. An average of the 3 scores was taken and used.(Jensen et al., 1994)(Childs et al., 2005) The Scale was measured at baseline evaluation, 1 week, 4 weeks and 6 months. In this study the Physical therapy intervention was performed for 4 weeks. No property measurement data were commented on in the article.

Selhorst GROC (Patient age 13 to 17 years of age)

Global Rating of Change (GROC) was used as a secondary measurement in the Selhorst article. This 15-point Likert type scale was used to assess perceived improvement (-7 to +7) A score of 0 represents no change from initial injury, +7 represents a great deal better, and -7 represents a great deal worse.(Jaeschke et al., 1989) The Scale was measured at baseline evaluation, 1 week, 4 weeks and 6 months. In this study the Physical therapy intervention was performed for 4 weeks. This GROC PCO has been validated for adult knee complaint.(Wang et al., 2011)

Walston-Modified Oswestry Disability Index (mODI) (Patient age 13 to 15 years of age)

The mODI was used as opposed to the Oswestry Disability to make the outcome appropriate for the pediatric population. One alteration was made to the index. "The mODI substitutes the question regarding sex with one about employment/home Making. (42 Hudson-Cook)" The mODI was self-reported and evaluated in the patients at evaluation and at discharge. "While the mODI has not been validated in the pediatric population, it has been used in previous studies to assess disability and outcomes, with mixed results.(7 Bronfort,81 Zapata)" (Walston & Yake, 2016)

Walston- Numeric Pain Scale (NPRS) (Patient age 13 to 15 years of age)

An 11-point NPRS was self-reported. The range used was from 0 (no pain) to 10 (maximal pain). This scale is based on patient perceived pain. The outcome was self-reported and evaluated in the patients at evaluation and at discharge. "level. It has been used successfully in the adult and pediatric populations to assess pain levels.(53 Maunuksela, 54 McCaffery) When assessing postoperative pain in pediatrics, the NPRS has demonstrated adequate reliability and concurrent validity.(49 LaMontagne, 61 O’Hara)"(Walston & Yake, 2016)

Walston-Fear Avoidance Behaviors Questionnaire (FABQ) (Patient age 13 to 15 years of age)

The FABQ looks at work (FABQ-W) and physical activity (FABQ-PA) subscales.

The outcome was self-reported and evaluated in the patients at evaluation and at discharge. "The FABQ-PA has demonstrated usefulness in assessing pediatric patients with chronic LBP.(80 Wilson) While the FABQ-PA would be a more appropriate measure of fear avoidance, the FABQ-W was used in this study because of its inclusion in the lumbar manipulation CPR criteria.(12 Childs, 28 Flynn, 29 Fritz)" (Walston & Yake, 2016)

Evans-LBP severity (primary outcome) (Patient age 12 to 18 years of age)

The patients in this study self-reported their low back pain severity using an 11-box numerical rating scale via questionnaire. Parameters were (0 for no pain, and 10 for the worst pain possible.) This PCO was the primary outcome in the study and was collected at the first 2 visits for baseline and again at 4, 8, 12, 26 and 52 weeks. 1."the 11-box numerical rating scale performs similarly to the visual analogue scale in adult and pediatric populations (Hjermstad)" (Evans et al., 2018)

Evans-18 item Roland-Morris Disability Questionnaire (Patient age 12 to 18 years of age)

This 18-item questionnaire was self-reported and used as a secondary measure and was collected at the first 2 visits for baseline and again at 4, 8, 12, 26 and 52 weeks. No property measurement data were mentioned in the article.

(Evans et al., 2018)

Evans- Quality of Life (23-item PedsQL) (Patient age 12 to 18 years of age)

A 23 item pediatric quality of life PCO was used as a secondary measure and was used as a secondary measure and was collected at the first 2 visits for baseline and again at 4, 8, 12, 26 and 52 weeks. No property measurement data were mentioned in the article. (Evans et al., 2018)

Evans- Improvement (Patient age 12 to 18 years of age)-collected from both patient and parent/caregiver

Improvement of the patient was used as a secondary measure and was collected at the first 2 visits for baseline and again at 4, 8, 12, 26 and 52 weeks. No property measurement data were mentioned in the article. (Evans et al., 2018)

Evans-Frequency of Medication use for low back pain (days/week) (Patient age 12 to 18 years of age)

Medication frequency(d/wk) being used by the patient specific for Low back pain was used as a secondary measure and was collected at the first 2 visits for baseline and again at 4, 8, 12, 26 and 52 weeks. No property measurement data were mentioned in the article. (Evans et al., 2018)

Evans- Satisfaction (Patient age 12 to 18 years of age)-collected from both patient and parent/caregiver

Patient satisfaction with care was used as a secondary measure and was collected at the first 2 visits for baseline and again at 4, 8, 12, 26 and 52 weeks. The 7-point scale used rating points of 1=completely satisfied, could not be better, and 7=completely dissatisfied, could not be worse.) a No property measurement data was mentioned in the article. Parents’ perception of patient improvement and satisfaction with care was asked as a secondary measurement in a questionnaire at 12, 26, and 52 weeks. No property measurement data were mentioned in the article.

(Evans et al., 2018)

**Cuboid Syndrome**

The Jennings article (Patient age 15 to 36 years of age) on Cuboid Syndrome used a Visual Analog Scale (VAS) pre and post treatment. The VAS used rated patients’ subjective pain from 0 (no pain) to 10 (worst pain imaginable). Property Measurements used in this article state that VAS for usual and worst pain are “reliable, valid, and responsive in assessing treatment outcome in persons with patellofemoral pain.” (Crossley et al., 2004). Another cited source within the article agrees that “the VAS is reliable , valid, and sensitive to treatment effects and easy to analyze.” (Salo et al., 2003).

**Structural (10 articles)**

Ten articles met the inclusion criteria for this category, including the following conditions: Scoliosis, Postural Asymmetry, Upper Cervical Dysfunction, Torticollis, Cranial Asymmetry, and Nonsynostotic Plagiocephaly. One Torticollis, One Nonsynostotic Plagiocephaly, One Cranial Asymmetry, and Three Scoliosis articles did not use any PCOs. One Postural Asymmetry, One Upper Cervical Dysfunction, and Two of the Scoliosis manuscripts did have the following PCOs.

**Scoliosis**

Rowe’s scoliosis article used the ***Scoliosis Quality of life (SQLI)*** (Rowe et al., 2006) (Patient age 10 to 16 years of age) as a secondary measure. property measurements of the SQLI were stated as being valid and reliable with a sufficient score distribution for a suggested patient population of ages 10 to 18 years of age (Feise et al., 2005; Rowe et al., 2006). SQLI was measured at baseline and at 6 months in this 6-month long study.

Morningstar used Functional Rating Index, VAS, and SF-36 were used but "...these results are not reported to avoid variability in outcome interpretation." (Morningstar et al., 2004)

**Postural Asymmetry**

Philippi’s RCT (Patient age 6 to 12 weeks of age) evaluating Postural Asymmetry, two questionnaires were used as secondary outcomes. One was a 3-point change questionnaire asking parents about changes in parameters: vomiting, sleeping, drinking, mood, excitability, stool frequency, and crying. No property measurements were discussed for this questionnaire. These measurements were taken at baseline and after each intervention.(Philippi et al., 2006)

**Upper Cervical Dysfunction**

Saedt’s Upper Cervical Dysfunction (Patient age less than 27 weeks of age) RCT used 3 PCO measures pre and post measurement. The measurements used were a study-specific Parent Questionnaire, Global Perceived Effect (GPE) Scale, and a VAS (Saedt et al., 2018) .The Parent Questionnaire gained information on symptoms and potential risk factors of the child (Gutmann, 2012) (Biedermann, 2005; Koch & Graumann-Brunt, 1999). The Global Perceived Effect (GPE) scale was used to capture the parent’s perception of improvement on a scale of 1 (very much improved/cured) to 7 (very much worse). (Hudak & Wright, 2000; Kamper et al., 2010; Saedt et al., 2018) VAS was used to assess symptom change over time, the scale was 0 (no change) to 100 (maximum change). These were collected at baseline and posttreatment. No property measurements of these PCO were discussed in this article. (Saedt et al., 2018)

**GI/GU (10 articles)**

Ten articles met the inclusion criteria for this category covering the following conditions: Five Infantile Colic, One Constipation and Cerebral Palsy, Two Suboptimal Infant Breastfeeding, One Pediatric Dysfunctional Voiding, and One Nocturnal Enuresis. All conditions included PCO data.

**Infantile Colic**

Miller (Patient age younger than 8 weeks)

In the Miller article, crying time from a 24-hour crying diary was monitored throughout the study period ending after 10 days or at discharge, whichever date came first. In studies of interventions for excessive crying in infants, there is no alternative to the outcome being based on the parent's self-report of crying behavior, and although crying diaries in themselves have been shown to be valid measures (Barr et al., 1988; Miller et al., 2012; St James-Roberts et al., 1993)

Browning (Patient age younger than 8 weeks)-Crying time diary was the primary outcome and the study used the mean number of crying episodes and the hours of sleep per day as secondary outcomes. The article stated that “The crying diary has been validated in previous studies”.(Barr et al., 1988; Kirjavainen et al., 2004). The diary was completed at the date of initial consultation and 14 days post 1st treatment.

“Four weeks after completion of the treatment trial the parents were interviewed by telephone as to whether there was complete resolution of colic symptoms (yes/no).”(Browning M, 2008)

Olafsdottir (Patient age 3 to 9 weeks)-Crying time diary is used in the Olafsdottir article. The 24-hour diary of infant's crying was filled out by Parent report at baseline and Effect of treatment was collected by phone interview 8 to 14 days after the date of last visit. Property measurement data were not mentioned in this article. Also used in this study, At each visit the parents described the effect of the last visit on a scale of five categories—“getting worse”, “no improvement”, “some improvement”, “marked improvement”, “completely well”—which were defined as the main outcome measure. One paediatrician (EO) was in contact with all the parents at each visit and filled in the scoring system.”(Olafsdottir et al., 2001)

Wiberg (Patient age 0 to 3 months)-Doctor Classification system based on parental report

retrospective study- notes were classified retrospectively. "Initially, classifications were carried out independently by 2 assessors, and thereafter, results were compared. In cases of different classification, information from data was discussed to reach an agreement of classification."(Wiberg & Wiberg, 2010) "it might also generate invalid evidence because the RCT is divorced from the real world and not necessarily externally valid.”(Bolton, 2001)

Hayden (Patient age 1 to 12 weeks of age)-24 hour Crying diary (parent reporting)

“Parents were also given a daily diary for use during the study; on this they recorded in every 24 h the amount of inconsolable crying, the total time spent sleeping, and the time the infant was being held or rocked (taken as an indication of low-level colic). Parents were asked to continue with bringing their infant to the clinic and completing the diary card even if the symptoms of colic resolved during the 4-week period.”(C. Hayden & Mullinger, 2006) ”The two main endpoints in this study were the mean number of hours/24 h spent with colicky crying and the mean number of hours/24 h spent sleeping.”(C. Hayden & Mullinger, 2006) The data was recorded in a Table in the article at Weeks 1,2,3, and 4 and a separate column calculating the change between weeks 1 and 4. No property measurements data were commented on in the article, however the author did state that uncontrollable crying “appears to be the most common variable measured.” (Barr et al., 1988; Carey, 1984; C. Hayden & Mullinger, 2006; WESSEL et al., 1954)

**Constipation and Cerebral Palsy**

Tarsuslu (Patient age 2 to 16 years of age)-Defecation Frequency was documented as times per week. “Initial assessment was conducted before the study, and follow-up evaluations were conducted at 3 and 6 months."(Tarsuslu et al., 2009) No property measurements data were commented on in the article.

Within the Tarsuslu other outcomes were used, however, they were used to measure the level of disability. (PCOs not included in these results-gross motor function classification system (explanatory variable), modified Ashworth scale, functional independence measure for children)

Constipation Assessment Scale (CAS)-The CAS used by Tarsuslu consists of 8 self-reported items investigating the presence and severity of constipation in both children and adults”.(Woolery et al., 2006) Those patients who were unable to self-report due to lack of comprehension due to age were allowed to submit parent and caregiver-reported results. (Tarsuslu et al., 2009) “The scoring is provided by a 3-point rating scale indicating 0 as no constipation, 1 as some problem, and 2 as severe problem. Total score ranges between 0 (no constipation) and 16 (severe constipation).”(Tarsuslu et al., 2009) “Initial assessment was conducted before the study, and follow-up evaluations were conducted at 3 and 6 months." (Tarsuslu et al., 2009) The CAS is reported in this article as a valid and reliable tool for both children and adults. (Tarsuslu et al., 2009; Woolery et al., 2006)

Visual Analog Scale (VAS)-A VAS was used in the Tarsuslu study to measure the satisfaction from treatment. This measurement was taken at 3 and 6 months. The VAS information was collected from the children and the parent/caregivers on scale from 0 cm (no satisfaction) to 10 cm (most satisfied) “VAS is most frequently used to evaluate the degree of pain, it has also been used to assess the burden and the severity of musculoskeletal disorders in various studies.30,31 Visual Analogue Scale use is shown to be a valid and reliable assessment method for use in children.32”(Angold et al., 1998; Bek et al., 2009; Stinson et al., 2006; Tarsuslu et al., 2009).

**Suboptimal Infant Breastfeeding**

Miller’s article (Patient age 2 days to 12 weeks), 2 PCOs were used, Mother’s report of exclusivity of breastfeeding and an improvement rating. In regard to the improvement rating, this was gathered by asking the mothers to rate improvement on a 10-point scale. The main result categories were partial improvement, no improvement or Worsening.(Miller et al., 2009) No further details about the PCOs were provided. No property measurements data were commented on in the article. The article states that "We did not use any of the common rating instruments as they have not been validated in any controlled manner." (R. T. Hall et al., 2002; Hazelbaker, 1993)

Vallone (Patient age “age at presentation” 1 day to 12 weeks)

Reported ability to latch and ability to breastfeed was the PCO used in the Vallone breastfeeding article. No specifics of the PCO were mentioned in the article, although it appears the data was collected during an extensive history. No property measurements data were commented on in the article. (S, 2004)

**Pediatric Dysfunctional Voiding (patient age 4-11)**

Nemett-Dysfunctional voiding symptoms were collected in this study as both objective and subjective measures. Subjective measures asked to either the parent or child about the number of days wet. The PCO data were gathered pre- and post- study treatment period, which lasted a minimum of 3 months. The pre-treatment symptoms were reported for at least 6 months before the start of the study. Using the pre- and post- PCO information, the days wet were categorized as: improved, same or worse. No property measurement data were mentioned in the article. (Nemett et al., 2008)

**Nocturnal Enuresis (patient age 3-18)**

Nemett-In the Nocturnal Enuresis study a weekly diary for wet night frequency was collected from the parents and child. Information was gathered at baseline, and 3, 6, 9, and 12 months after the commencement of the study. The baseline data were from a 2-week diary noting the number of wet nights, which was said to be “consistent with other baseline measurements reported in the literature.”(Nemett et al., 2008; van Poecke & Cunliffe, 2009) The weekly diary also noted “possible associated factors” (i.e., parties, birthdays, other stressful/emotional occurrences). No other property measurement data were mentioned in the article.

**Special Needs (10 articles)**

Ten articles met the inclusion criteria for this category covering the following conditions: Autism (2), Attention Deficit/Hyperactivity Disorder (1), Cerebral Palsy (3), Preterm Infants (4). Two Autism, Three Cerebral Palsy Articles did contain PCOs. Four Preterm Infant and One Attention Deficit/Hyperactivity Disorder studies did not contain Patient Centered outcomes. All other conditions in this category included PCO data and have been included in this section.

**Autism**

Khorshid (Patient age 4 to 16 years of age) -Autism Treatment Evaluation Checklist (ATEC) was taken at the start of the study and then monthly throughout treatment. The ATEC is given in Khorshid Table 1. This tool by Rimland and Edelson by the Autism Research Institute includes 4 sections: speech, sociability, sensory/cognitive awareness, health/physical/ behavior with separate scale qualifications given at the start of each sections questions. (Khorshid KA, Sweat RW, Zemba D, 2006) This PCO is “completed by parents to monitor the clinical changes in their autistic children. ((Khorshid KA, Sweat RW, Zemba D, 2006)It includes The checklist is available in Table 1 of the Khorshid article. [www.autism.com/atec](http://www.autism.com/atec). On this website, the Autism Research Institute states that “studies confirm validity of ATEC” and that studies on reliability are underway. ([www.autism.com/atec](http://www.autism.com/atec)) (Sturmey, 2005)

Bramati-Casterllarin (Patient age 3.5 to 8 years of age)-“A modified standardised Autism Research Institute -S.O.S questionnaire was used to assess behavioural and gastrointestinal symptoms structured as a 24 parameter grid, with four sub-scales; social behaviour, ritual and repetitive activities, digestive symptoms, and general symp

toms” (Bramati-Castellarin et al., 2016; Brudnak et al., 2002; Erickson et al., 2005; Esch & Carr, 2004; Sturmey, 2005; Williams et al., 2005) (Rimland, 1998)

The parent observed changes in behavior Using the “Autism Research Institute Secretin Outcomes Survey Form ('S.O.S. Form') (Bramati-Castellarin et al., 2016)

The study consisted of three periods, Period I - control(baseline), Period II - treatment, and Period III - posttreatment. The outcome measures for the study were the nine completed S.O.S questionnaires used to assess GI and behavioural signs and symptoms during Periods I, II and III of the study.” During Period 1 of the study 4 total questionnaires were completed at weeks 1,3,5, and 6. These were labeled Questionnaires 1-4. During Period 2 of the study 4 total questionnaires were completed at weeks 8,9,10 and 12. These were labeled Questionnaires 5-8. During Period 3 of the study 1 total questionnaire was completed at week 18. This was labeled Questionnaire 9. (Bramati-Castellarin et al., 2016)

The article states that this is a validated questionnaire. (Bramati-Castellarin et al., 2016)

**Cerebral Palsy**

Wyatt (Patient age 5 to 12 years of age)-Quality of life using Child Health Questionnaire (CHQ) PF50 (Landgraf JM , Aberz L, 1996; Wyatt et al., 2011) primary outcome at baseline, 10 weeks, and 6 months after study entry. The CHQ was completed by the parent/carer as a “generic measure of the children’s quality of life.” (Wyatt et al., 2011) In Table 1, the author included the outcome measure description. For this primary outcome, subscales used were the Family activities subscale(CHQ-FA), the Family cohesion subscale (CHQ-FC), Physical Summary Score (CHQ-PhS), and Psychological Summary Score (CHQ-PsS). In the CHQ-FC the score ranges from 0-100 where the higher score indicates better family functioning. In the CHQ-PsS, the higher score indicates better health. The article states that this outcome is used widely in children with Cerebral Palsy. (Majnemer et al., 2007)(McCarthy et al., 2002; Wake et al., 2003) No property measurement data were mentioned in the article other than its wide use in children with Cerebral Palsy as stated above.

Wyatt (Patient age 5 to 12 years of age)-Parent assessment of child global health and sleep (Wyatt et al., 2011)secondary outcome at 10 weeks and 6 months

This outcome was completed by the parent/carer. Parents were asked to make a simple global assessment of whether they rated their children’s overall health and wellbeing as the ‘same’, ‘better’ or ‘worse’ than when they entered the study and the same overall assessment of children’s sleeping. For the purposes of the statistical analyses, the Global Health outcomes were categorized as ‘better’ versus ‘same’ or ‘worse’. No property measurement data were mentioned in the article

Wyatt (Patient age 5 to 12 years of age)-Pediatric Pain Profile (PPP) (Hunt et al., 2004; Wyatt et al., 2011)- secondary outcome at baseline, 10 weeks and 6 months. (Wyatt et al., 2011)

This outcome was completed by the parent/carer. It is a “A behaviour rating scale designed and validated specifically for children with disabilities. Children were rated on their ‘best’ and ‘worst’ day in terms of their pain over the preceding week.” Each was assigned a ‘best day’ pain score and a ‘worst day’ pain score. The wording for the ‘worst day’ score was “The most painful episode of the most troublesome pain for your child in the last week” ((Wyatt et al., 2011)Table 1) The score range was 0-60 with the higher score indicating the worst pain. No property measurement data were mentioned in the article.(Hunt et al., 2004)

Wyatt (Patient age 5 to 12 years of age)-Fit and sleep diaries and Global Health and Sleeping - secondary outcome at baseline, 10 weeks and 6 months (Wyatt et al., 2011)

The child’s global health and sleeping was measured by indicating whether the child’s general health and sleeping was same/better/worse than previously. The fit and sleep were noted in a diary. The sleeping measure, recorded in a 7 day diary and adapted by a diary used by Montgomery. (Montgomery et al., 2004) allows recording of both time to settle and time spent asleep. In the same diary, the parent/caregiver noted the number of fits experienced by the child. Sleep was measured in both the mean score it took the child to fall asleep and the mean score of how long the child slept overnight. This outcome was completed by the parent/carer.(Montgomery et al., 2004)No property measurement data were mentioned in the article.

Wyatt (Patient age 5 to 12 years of age)-Carer/Parent Quality of life taken by Short Form-36 (SF-36) of main carer (Short Form 36)(Wyatt et al., 2011) secondary outcome at baseline, 10 weeks and 6 months. This outcome was completed by the parent/carer.(JE, 1993) The subscales noted as used are the Physical Component Summary Score (SF-36 PCS) and the Mental Component Summary Score (SF-36 MCS). The scores taken have a population mean of 50. The study assigned any score above 50 as better health and any score below 50 as poorer health. No property measurement data were mentioned in the article, although Table 1 states that the SF-36 is the “most widely used generic measure of adult quality of life.(Wyatt et al., 2011)

The Wyatt study also included the objective outcome, Gross Motor Function Measure-66. (Wyatt et al., 2011)

Duncan 2004 (Patient age 11 months to 12 years of age)-In the Duncan article, a parent interview including 2 visual log scales was used at the conclusion of the study to gauge the parents’ perceptions of the changes they perceived in their child during the course of the entire study. “The parents were simply asked an open-ended question: “Did you note any changes in your child as a result of the therapies, and if so, what were the changes?”

In addition to the interview question, parents were also asked to mark their perception of their child’s level of muscle stiffness and their child’s level of happiness on 2 separate visual log scales, 100 millimeters in length. The study then included specific comments made by parents during the interview process in the results section. In Table 2, the author listed areas of improvement by parents. The areas of improvement noted were: Leg or hand use, sleep, improved mood, worsened mood, speech or drooling, bowel movements, and cognition. No property measurement data were mentioned in the article. (B Duncan et al., 2004)

Duncan 2008 (Patient age 20 months to 12 years of age)-In the 2008 Duncan article, a VAS was administered to parents to rate spasticity of the child. The parents were asked to rate the child’s arched back and startle reflex using a scale from 0 (least severe) to 100 (most severe). They were asked to rate the current spasticity compared to the usual level of spasticity. The VAS was completed at baseline, midway through the 24 weeks of treatment, and at the conclusion of the treatments. The article used several objective outcomes. No property measurement data were mentioned in the article for the VAS used. (Burris Duncan et al., 2008)

**Respiratory (8 articles)**

Respiratory conditions that were identified as meeting the inclusion criteria for this study were Asthma, Otitis Media, and Obstructive Apnea. Eight articles in total met the inclusion criteria. Two Articles on Asthma and Two articles on Otitis Media contained PCOs. One article on Obstructive Apnea, one article on asthma, and two Otitis Media articles did not contain PCOs. The article on Obstructive Apnea only used objective measures, so not included in this review.

**Asthma**

Two Asthma articles were included: one by Bronfort and one by Guiney. The Guiney article did not include a PCO. The Bronfort Article (Patient age 6 to 17 years of age) did use two PCOS, a Quality of Life Questionnaire and a PCO based on Asthma severity and improvement. Only one of the two asthma RCTs used a PCO

Bronfort et al. administered the Quality of Life Questionnaire as both an interviewer and self-reported questionnaire. Asthma severity scale from 0 (no symptoms) to 10 (worst symptoms) was assessed by patient and parent. A 9-point improvement scale from ‘no symptoms:100% better’ to ‘twice as bad:100% worse’ was also asked to both patient and parent/guardian (Bronfort et al., 2001; Juniper et al., 1996b, 1996a) All questionnaires were assessed at baseline and after 12 weeks of treatment, but the severity and improvement questionnaire was also administered by postal services 1 year after the end of treatment. Property measurements of the asthma severity and improvement questionnaire were not mentioned in the article. (Bronfort et al., 2001)

**Otitis Media**

Four articles were evaluated for PCO use in the condition of Otitis Media (OM); however only 2 used PCOs.

Degenhardt et al. RCT (Patient age 7 months to 35 months) used parent-reported occurrence of OM by a telephone interview to document the number of middle ear infections for the child participant. The occurrence of OM was assessed at baseline during a health history and then one year after the 3-week intervention period to collect data on the number of the “number of middle ear infections that each subject had since intervention.”(Degenhardt & Kuchera, 2006)Property measurements for parent reported occurrences of OM were not mentioned in the article.

Mills et al. RCT (Patient age 6 months to 6 years old) on acute otitis media used one PCO that was conducted by the study coordinator to obtain medical history regarding medications, illnesses, and injuries, as well as a 5-point scale (5=“much more”;1=“much less”) to assess behavioral changes. These behaviors were irritability, disobedience, ear pulling, appetite, restful sleep, hearing when spoken to, listening to conversation, talking, and clumsiness, which were combined into the following categories: hearing (hearing when spoken to, listening to conversation, and talking), negative behaviors (irritability, disobedience, and ear pulling), or positive behaviors (sleeping and appetite). This measurement was gathered at baseline and at each visit by the site coordinator. No property measurements were mentioned regarding this PCO. (Mills et al., 2003)

At each visit, the site coordinator obtained interval history about medications, illnesses, injuries, and changes in behavior by using a 5-point scale (5=“much more”;1=“much less” in comparison with behavior in other children) to rate irritability, disobedience, ear pulling, appetite, restful sleep, hearing when spoken to, listening to conversation, talking, and clumsiness. (Mills et al., 2003)

**Bibliography**

Angold, A., Messer, S. C., Stangl, D., Farmer, E. M., Costello, E. J., & Burns, B. J. (1998). Perceived parental burden and service use for child and adolescent psychiatric disorders. *American Journal of Public Health*, *88*(1), 75–80. https://doi.org/10.2105/AJPH.88.1.75

Barr, R. G., Kramer, M. S., Boisjoly, C., McVey-White, L., & Pless, I. B. (1988). Parental diary of infant cry and fuss behaviour. *Archives of Disease in Childhood*, *63*(4), 380 LP – 387. https://doi.org/10.1136/adc.63.4.380

Bek, N., Simsek, I. E., Erel, S., Yakut, Y., & Uygur, F. (2009). Turkish version of impact on family scale: a study of reliability and validity. *Health and Quality of Life Outcomes*, *7*(1), 4. https://doi.org/10.1186/1477-7525-7-4

Biedermann, H. (2005). Manual therapy in children: proposals for an etiologic model. *Journal of Manipulative and Physiological Therapeutics*, *28*(3), e1-15. https://doi.org/10.1016/j.jmpt.2005.02.011

Bolton, J. E. (2001). The evidence in evidence-based practice: what counts and what doesn’t count? In *Journal of manipulative and physiological therapeutics* (Vol. 24, Issue 5, pp. 362–366). https://doi.org/10.1067/mmt.2001.115259

Borusiak, P., Biedermann, H., Bosserhoff, S., & Opp, J. (2010). Lack of efficacy of manual therapy in children and adolescents with suspected cervicogenic headache: results of a prospective, randomized, placebo-controlled, and blinded trial. *Headache*, *50*(2), 224–230. https://doi.org/10.1111/j.1526-4610.2009.01550.x

Bramati-Castellarin, I., Patel, V. B., & Drysdale, I. P. (2016). Repeat-measures longitudinal study evaluating behavioural and gastrointestinal symptoms in children with autism before, during and after visceral osteopathic technique (VOT). *Journal of Bodywork and Movement Therapies*, *20*(3), 461–470. https://doi.org/10.1016/j.jbmt.2016.01.001

Bronfort, G., Evans, R. L., Kubic, P., & Filkin, P. (2001). Chronic pediatric asthma and chiropractic spinal manipulation: a prospective clinical series and randomized clinical pilot study. *Journal of Manipulative and Physiological Therapeutics*, *24*(6), 369–377. https://doi.org/10.1067/mmt.2001.116417

Browning M, M. J. (2008). . Comparison of the short-term effects of chiropractic spinal manipulation and occipito-sacral decompression in the treatment of infant colic: a single-blinded, randomized, comparison trial. *Clin Chiropr.*, *11*(3), 122–129.

Brudnak, M. A., Rimland, B., Kerry, R. E., Dailey, M., Taylor, R., Stayton, B., Waickman, F., Waickman, M., Pangborn, J., & Buchholz, I. (2002). Enzyme-based therapy for autism spectrum disorders – Is it worth another look? *Medical Hypotheses*, *58*(5), 422–428. https://doi.org/https://doi.org/10.1054/mehy.2001.1513

Carey, W. B. (1984). “Colic”—Primary Excessive Crying as an Infant-Environment Interaction. *Pediatric Clinics of North America*, *31*(5), 993–1005. https://doi.org/https://doi.org/10.1016/S0031-3955(16)34681-8

Childs, J. D., Piva, S. R., & Fritz, J. M. (2005). Responsiveness of the Numeric Pain Rating Scale in Patients with Low Back Pain. *Spine*, *30*(11). https://journals.lww.com/spinejournal/Fulltext/2005/06010/Responsiveness_of_the_Numeric_Pain_Rating_Scale_in.18.aspx

Crossley, K. M., Bennell, K. L., Cowan, S. M., & Green, S. (2004). Analysis of outcome measures for persons with patellofemoral pain: which are reliable and valid?11No commercial party having a direct financial interest in the results of the research supporting this article has or will confer a benefit upon the author(s). *Archives of Physical Medicine and Rehabilitation*, *85*(5), 815–822. https://doi.org/https://doi.org/10.1016/S0003-9993(03)00613-0

Degenhardt, B. F., & Kuchera, M. L. (2006). Osteopathic evaluation and manipulative treatment in reducing the morbidity of otitis media: a pilot study. *The Journal of the American Osteopathic Association*, *106*(6), 327–334.

Duncan, B, Barton, L., Edmonds, D., & Blashill, B. M. (2004). Parental perceptions of the therapeutic effect from osteopathic manipulation or acupuncture in children with spastic cerebral palsy. *Clinical Pediatrics*, *43*(4), 349–353. https://doi.org/10.1177/000992280404300406

Duncan, Burris, McDonough-Means, S., Worden, K., Schnyer, R., Andrews, J., & Meaney, F. J. (2008). Effectiveness of osteopathy in the cranial field and myofascial release versus acupuncture as complementary treatment for children with spastic cerebral palsy: a pilot study. *The Journal of the American Osteopathic Association*, *108*(10), 559–570.

Erickson, C. A., Stigler, K. A., Corkins, M. R., Posey, D. J., Fitzgerald, J. F., & McDougle, C. J. (2005). Gastrointestinal Factors in Autistic Disorder: A Critical Review. *Journal of Autism and Developmental Disorders*, *35*(6), 713. https://doi.org/10.1007/s10803-005-0019-4

Esch, B. E., & Carr, J. E. (2004). Secretin as a Treatment for Autism: A Review of the Evidence. *Journal of Autism and Developmental Disorders*, *34*(5), 543–556. https://doi.org/10.1007/s10803-004-2549-6

Evans, R., Haas, M., Schulz, C., Leininger, B., Hanson, L., & Bronfort, G. (2018). Spinal manipulation and exercise for low back pain in adolescents: a randomized trial. *Pain*, *159*(7), 1297–1307. https://doi.org/10.1097/j.pain.0000000000001211

Feise, R. J., Donaldson, S., Crowther, E. R., Menke, J. M., & Wright, J. G. (2005). Construction and Validation of the Scoliosis Quality of Life Index in Adolescent Idiopathic Scoliosis. *Spine*, *30*(11). https://journals.lww.com/spinejournal/Fulltext/2005/06010/Construction_and_Validation_of_the_Scoliosis.15.aspx

Fritz, J. M., Childs, J. D., & Flynn, T. W. (2005). Pragmatic application of a clinical prediction rule in primary care to identify patients with low back pain with a good prognosis following a brief spinal manipulation intervention. *BMC Family Practice*, *6*(1), 29. https://doi.org/10.1186/1471-2296-6-29

Gutmann, G. (2012). Das cervical-diencephal-statische Syndrom des Kleinkindes. *Manuelle Medizin*, *50*(4), 277–284. https://doi.org/10.1007/s00337-012-0918-3

Hall, A. M., Maher, C. G., Latimer, J., Ferreira, M. L., & Costa, L. O. P. (2011). The patient-specific functional scale is more responsive than the Roland Morris disability questionnaire when activity limitation is low. *European Spine Journal*, *20*(1), 79–86. https://doi.org/10.1007/s00586-010-1521-8

Hall, R. T., Mercer, A. M., Teasley, S. L., McPherson, D. M., Simon, S. D., Santos, S. R., Meyers, B. M., & Hipsh, N. E. (2002). A breast-feeding assessment score to evaluate the risk for cessation of breast-feeding by 7 to 10 days of age. *The Journal of Pediatrics*, *141*(5), 659–664. https://doi.org/https://doi.org/10.1067/mpd.2002.129081

Hayden, C., & Mullinger, B. (2006). A preliminary assessment of the impact of cranial osteopathy for the relief of infantile colic. *Complementary Therapies in Clinical Practice*, *12*(2), 83–90. https://doi.org/10.1016/j.ctcp.2005.12.005

Hayden, J. A., Mior, S. A., & Verhoef, M. J. (2003). Evaluation of chiropractic management of pediatric patients with low back pain: a prospective cohort study. *Journal of Manipulative and Physiological Therapeutics*, *26*(1), 1–8. https://doi.org/10.1067/mmt.2003.11

Hazelbaker, A. (1993). The assessment tool for lingual frenulum function (ATLFF): Use in a lactation consultant private practice. *Pacific Oaks College*.

Hudak, P. L., & Wright, J. G. (2000). The Characteristics of Patient Satisfaction Measures. *Spine*, *25*(24). https://journals.lww.com/spinejournal/Fulltext/2000/12150/The_Characteristics_of_Patient_Satisfaction.12.aspx

Hunt, A., Goldman, A., Seers, K., Crichton, N., Mastroyannopoulou, K., Moffat, V., Oulton, K., & Brady, M. (2004). Clinical validation of the Paediatric Pain Profile. *Developmental Medicine & Child Neurology*, *46*(01), 9–18. https://doi.org/10.1017/S0012162204000039

Jaeschke, R., Singer, J., & Guyatt, G. H. (1989). Measurement of health status. Ascertaining the minimal clinically important difference. *Controlled Clinical Trials*, *10*(4), 407–415. https://doi.org/10.1016/0197-2456(89)90005-6

JE, W. (1993). SF-36 Health Survey: manual and interpretation guide. *Boston: The Health Institute, New England Medical Centre*.

Jensen, M. P., Turner, J. A., & Romano, J. M. (1994). What is the maximum number of levels needed in pain intensity measurement? *Pain*, *58*(3), 387–392. https://doi.org/https://doi.org/10.1016/0304-3959(94)90133-3

Juniper, E. F., Guyatt, G. H., Feeny, D. H., Ferrie, P. J., Griffith, L. E., & Townsend, M. (1996a). Measuring quality of life in children with asthma. *Quality of Life Research*, *5*(1), 35–46. https://doi.org/10.1007/BF00435967

Juniper, E. F., Guyatt, G. H., Feeny, D. H., Ferrie, P. J., Griffith, L. E., & Townsend, M. (1996b). Measuring quality of life in the parents of children with asthma. *Quality of Life Research*, *5*(1), 27–34. https://doi.org/10.1007/BF00435966

Kamper, S. J., Ostelo, R. W. J. G., Knol, D. L., Maher, C. G., de Vet, H. C. W., & Hancock, M. J. (2010). Global Perceived Effect scales provided reliable assessments of health transition in people with musculoskeletal disorders, but ratings are strongly influenced by current status. *Journal of Clinical Epidemiology*, *63*(7), 760-766.e1. https://doi.org/https://doi.org/10.1016/j.jclinepi.2009.09.009

Khorshid KA, Sweat RW, Zemba D, Z. B. (2006). Clinical efficacy of upper cervical versus full spine chiropractic care on children with autism: a randomized clinical trial. *J Vertebral Subluxation Res*, 1–7.

Kirjavainen, J., Lehtonen, L., Kirjavainen, T., & Kero, P. (2004). Sleep of Excessively Crying Infants: A 24-Hour Ambulatory Sleep Polygraphy Study. *Pediatrics*, *114*(3), 592 LP – 600. https://doi.org/10.1542/peds.2003-0651-L

Koch, L. E., & Graumann-Brunt, S. (1999). Kopfgelenk-induzierte Symmetriestörungen und deren FolgepathologienPilot-Studie. *Manuelle Medizin*, *37*(2), 67–77. https://doi.org/10.1007/s003370050107

Landgraf JM , Aberz L, W. J. (1996). The CHQ user’s manual . *Boston: The Health Institute, New England Medical Centre*, *1st editio*.

Majnemer, A., Shevell, M., Rosenbaum, P., Law, M., & Poulin, C. (2007). Determinants of Life Quality in School-Age Children with Cerebral Palsy. *The Journal of Pediatrics*, *151*(5), 470-475.e3. https://doi.org/https://doi.org/10.1016/j.jpeds.2007.04.014

McCarthy, M. L., Silberstein, C. E., Atkins, E. A., Harryman, S. E., Sponseller, P. D., & Hadley-Miller, N. A. (2002). Comparing reliability and validity of pediatric instruments for measuring health and well-being of children with spastic cerebral palsy. *Developmental Medicine & Child Neurology*, *44*(07), 468–476. https://doi.org/10.1017/S0012162201002377

Miller, J. E., Miller, L., Sulesund, A.-K., & Yevtushenko, A. (2009). Contribution of chiropractic therapy to resolving suboptimal breastfeeding: a case series of 114 infants. *Journal of Manipulative and Physiological Therapeutics*, *32*(8), 670–674. https://doi.org/10.1016/j.jmpt.2009.08.023

Miller, J. E., Newell, D., & Bolton, J. E. (2012). Efficacy of chiropractic manual therapy on infant colic: a pragmatic single-blind, randomized controlled trial. *Journal of Manipulative and Physiological Therapeutics*, *35*(8), 600–607. https://doi.org/10.1016/j.jmpt.2012.09.010

Mills, M. V, Henley, C. E., Barnes, L. L. B., Carreiro, J. E., & Degenhardt, B. F. (2003). The use of osteopathic manipulative treatment as adjuvant therapy in children with recurrent acute otitis media. *Archives of Pediatrics & Adolescent Medicine*, *157*(9), 861–866. https://doi.org/10.1001/archpedi.157.9.861

Montgomery, P., Stores, G., & Wiggs, L. (2004). The relative efficacy of two brief treatments for sleep problems in young learning disabled (mentally retarded) children: a randomised controlled trial. *Archives of Disease in Childhood*, *89*(2), 125 LP – 130. https://doi.org/10.1136/adc.2002.017202

Morningstar, M. W., Woggon, D., & Lawrence, G. (2004). Scoliosis treatment using a combination of manipulative and rehabilitative therapy: a retrospective case series. *BMC Musculoskeletal Disorders*, *5*, 32. https://doi.org/10.1186/1471-2474-5-32

Nemett, D. R., Fivush, B. A., Mathews, R., Camirand, N., Eldridge, M. A., Finney, K., & Gerson, A. C. (2008). A randomized controlled trial of the effectiveness of osteopathy-based manual physical therapy in treating pediatric dysfunctional voiding. *Journal of Pediatric Urology*, *4*(2), 100–106. https://doi.org/10.1016/j.jpurol.2007.11.006

Olafsdottir, E., Forshei, S., Fluge, G., & Markestad, T. (2001). Randomised controlled trial of infantile colic treated with chiropractic spinal manipulation. *Archives of Disease in Childhood*, *84*(2), 138–141. https://doi.org/10.1136/adc.84.2.138

Philippi, H., Faldum, A., Schleupen, A., Pabst, B., Jung, T., Bergmann, H., Bieber, I., Kaemmerer, C., Dijs, P., & Reitter, B. (2006). Infantile postural asymmetry and osteopathic treatment: a randomized therapeutic trial. *Developmental Medicine and Child Neurology*, *48*(1), 5–9; discussion 4. https://doi.org/10.1017/S001216220600003X

Przekop, P., Przekop, A., & Haviland, M. G. (2016). Multimodal compared to pharmacologic treatments for chronic tension-type headache in adolescents. *Journal of Bodywork and Movement Therapies*, *20*(4), 715–721. https://doi.org/10.1016/j.jbmt.2015.02.003

Rimland. (1998). Unlocking the Potential of Secretin The Autism Research Institute-San Diego CA. *Autism Research Institute*.

Rowe, D. E., Feise, R. J., Crowther, E. R., Grod, J. P., Menke, J. M., Goldsmith, C. H., Stoline, M. R., Souza, T. A., & Kambach, B. (2006). Chiropractic manipulation in Adolescent Idiopathic Scoliosis: a pilot study. *Chiropractic & Osteopathy*, *14*(1), 15. https://doi.org/10.1186/1746-1340-14-15

S, V. (2004). Chiropractic evaluation and treatment of musculoskeletal dysfunction in infants demonstrating difficulty breastfeeding. *J Clin Chiropr Ped*, *6*(1), 349–368.

Saedt, E. R. I. C., Driehuis, F., Hoogeboom, T. J., van der Woude, B. H., de Bie, R. A., & Nijhuis-van der Sanden, M. W. G. (2018). Common Manual Therapy Practices in the Netherlands for Infants With Upper Cervical Dysfunction: A Prospective Cohort Study. *Journal of Manipulative and Physiological Therapeutics*, *41*(1), 52–61. https://doi.org/10.1016/j.jmpt.2017.08.003

Salo, D., Eget, D., Lavery, R. F., Garner, L., Bernstein, S., & Tandon, K. (2003). Can patients accurately read a visual analog pain scale? *The American Journal of Emergency Medicine*, *21*(7), 515–519. https://doi.org/https://doi.org/10.1016/j.ajem.2003.08.022

Selhorst, M., & Selhorst, B. (2015). Lumbar manipulation and exercise for the treatment of acute low back pain in adolescents: a randomized controlled trial. *The Journal of Manual & Manipulative Therapy*, *23*(4), 226–233. https://doi.org/10.1179/2042618614Y.0000000099

St James-Roberts, I., Hurry, J., & Bowyer, J. (1993). Objective confirmation of crying durations in infants referred for excessive crying. *Archives of Disease in Childhood*, *68*(1), 82 LP – 84. https://doi.org/10.1136/adc.68.1.82

Staes, F., Stappaerts, K., Vertommen, H., & Nuyens, G. (2000). Visual analogue scale for the perceived influence of exertion and movements/positions on low back problems in surveys of adolescents. *Acta Paediatrica*, *89*(6), 713–716. https://doi.org/10.1111/j.1651-2227.2000.tb00371.x

Stinson, J. N., Kavanagh, T., Yamada, J., Gill, N., & Stevens, B. (2006). Systematic review of the psychometric properties, interpretability and feasibility of self-report pain intensity measures for use in clinical trials in children and adolescents. *Pain*, *125*(1), 143–157. https://doi.org/https://doi.org/10.1016/j.pain.2006.05.006

Sturmey, P. (2005). Secretin is an ineffective treatment for pervasive developmental disabilities: a review of 15 double-blind randomized controlled trials. *Research in Developmental Disabilities*, *26*(1), 87–97. https://doi.org/https://doi.org/10.1016/j.ridd.2004.09.002

Tarsuslu, T., Bol, H., Simşek, I. E., Toylan, I. E., & Cam, S. (2009). The effects of osteopathic treatment on constipation in children with cerebral palsy: a pilot study. *Journal of Manipulative and Physiological Therapeutics*, *32*(8), 648–653. https://doi.org/10.1016/j.jmpt.2009.08.016

van Poecke, A. J., & Cunliffe, C. (2009). Chiropractic treatment for primary nocturnal enuresis: a case series of 33 consecutive patients. *Journal of Manipulative and Physiological Therapeutics*, *32*(8), 675–681. https://doi.org/10.1016/j.jmpt.2009.08.019

Varni, J. W., Thompson, K. L., & Hanson, V. (1987). The Varni/Thompson Pediatrie Pain Questionnaire. I. Chronic musculoskeletal pain in juvenile rheumatoid arthritis. *Pain*, *28*(1), 27–38. https://doi.org/https://doi.org/10.1016/0304-3959(87)91056-6

Wake, M., Salmon, L., & Reddihough, D. (2003). Health status of Australian children with mild to severe cerebral palsy: cross-sectional survey using the Child Health Questionnaire. *Developmental Medicine & Child Neurology*, *45*(03), 194–199. https://doi.org/10.1017/S0012162203000379

Walston, Z., & Yake, D. (2016). Lumbar Thrust Manipulation and Exercise for the Treatment of Mechanical Low Back Pain in Adolescents: A Case Series. *The Journal of Orthopaedic and Sports Physical Therapy*, *46*(5), 391–398. https://doi.org/10.2519/jospt.2016.6366

Wang, Y.-C., Hart, D. L., Stratford, P. W., & Mioduski, J. E. (2011). Baseline Dependency of Minimal Clinically Important Improvement. *Physical Therapy*, *91*(5), 675–688. https://doi.org/10.2522/ptj.20100229

Ware Jr. J.E. Kosinski., Dewey, J. . (2002). HOw to Score Version 2 of the SF-12 Health Survey. *Quality Metric, Inc, Lincoln, RI.*

WESSEL, M. A., COBB, J. C., JACKSON, E. B., HARRIS, G. S., & DETWILER, A. N. N. C. (1954). PAROXYSMAL FUSSING IN INFANCY, SOMETIMES CALLED &quot;COLIC&quot; *Pediatrics*, *14*(5), 421 LP – 435. http://pediatrics.aappublications.org/content/14/5/421.abstract

Wiberg, K. R., & Wiberg, J. M. M. (2010). A retrospective study of chiropractic treatment of 276 danish infants with infantile colic. *Journal of Manipulative and Physiological Therapeutics*, *33*(7), 536–541. https://doi.org/10.1016/j.jmpt.2010.08.004

Williams, K. J., Wray, J. J., & Wheeler, D. M. (2005). Intravenous secretin for autism spectrum disorder. In K. J. Williams (Ed.), *Cochrane Database of Systematic Reviews* (Issue 3). John Wiley & Sons, Ltd. https://doi.org/10.1002/14651858.CD003495.pub2

Woolery, M., Carroll, E., Fenn, E., Wieland, H., Jarosinski, P., Corey, B., & Wallen, G. R. (2006). A Constipation Assessment Scale for Use in Pediatric Oncology. *Journal of Pediatric Oncology Nursing*, *23*(2), 65–74. https://doi.org/10.1177/1043454205285874

Wyatt, K., Edwards, V., Franck, L., Britten, N., Creanor, S., Maddick, A., & Logan, S. (2011). Cranial osteopathy for children with cerebral palsy: a randomised controlled trial. *Archives of Disease in Childhood*, *96*(6), 505–512. https://doi.org/10.1136/adc.2010.199877
