# Supplementary material for: Patient-centered outcomes used in pediatric focused manual therapies research studies: a secondary data analysis of a systematic review
Source: J Patient Rep Outcomes. 2021 Apr 1;5:31. doi: 10.1186/s41687-021-00305-1 (PMC8017068; doi:10.1186/s41687-021-00305-1)
Supplement: Supplementary file 2 — Additional file 2. [file 41687_2021_305_MOESM2_ESM.docx]

Supplement #2

COSMIN Analysis Results

| **COSMIN Category** | **Box** | **Assessments Performed** |
| --- | --- | --- |
| Internal Consistency | A | 2 |
| Reliability | B | 3 |
| Measurement Error | C | 0 |
| Content Validity | D | 3 |
| Structural Validity | E | 4 |
| Hypothesis Validity | F | 0 |
| Cross-Cultural Validity (we used the term "Construct Validity") | G | 0 |
| Criterion Validity | H | 4 |
| Responsiveness | I | 1 |
| Total Cosmin Assessments Performed |  | 17 |
|  |  |  |
| 9 COSMIN measurement properties (categories) included in COSMIN analysis |  |  |
| 3 were not applicable to our PCOs |  |  |
| 6 were applicable to at least 1 PCO-each has a tab in this spreadsheet |  |  |

| **Reliability - Box B** |  |  |  |  |  |  |  |  |  |  |  |  |  |  |  |  |  |  |
| --- | --- | --- | --- | --- | --- | --- | --- | --- | --- | --- | --- | --- | --- | --- | --- | --- | --- | --- |
| PCO | Condition | Original Article | Reference Article | 1 | 2 | 3 | 4 | 5 | 6 | 7 | 8 | 9 | 10 | 11 | 12 | 13 | 14 | Overall Score |
| Self-report VAS | LBP | Hayden 2003 | Staes | Excellent | Fair | Good | Excellent | Excellent | Excellent | Fair | Excellent | Fair | Excellent | N/A | Excellent | Excellent | Excellent | **FAIR** |
| VAS | Cuboid | Jennings 2005 | Crossley | Good | Fair | Poor | Excellent | Good | Excellent | Good | Excellent | Excellent | Poor | Excellent | N/A | N/A | N/A | **POOR** |
| 5. CAS (Constipation Assessment scale) | Constipation and cerebral palsy | Tarsuslu | Woolery | Good | Fair | Poor | Excellent | Fair | Excellent | Fair | Excellent | Excellent | Fair | N/A | N/A | Poor | Good | **POOR** |

| **Internal Consistency - Box A** |  |  |  |  |  |  |  |  |  |  |  |  |  |  |  |
| --- | --- | --- | --- | --- | --- | --- | --- | --- | --- | --- | --- | --- | --- | --- | --- |
| PCO | Condition | Original Article | Reference Article | 1 | 2 | 3 | 4 | 5 | 6 | 7 | 8 | 9 | 10 | 11 | Overall Score |
| FABQ | Mechanical LBP | Walston | Wilson | Yes | Good | Fair | Fair | Poor | Fair | Excellent | Fair | Excellent | Excellent | N/A | **POOR** |
| ATEC (Autism Treatment Evaluation Checklist) | Autism | Khorshid | Magiati | Yes | Good | Fair | Poor | Fair | Poor | Excellent | Fair | Excellent | Excellent | Poor | **POOR** |

| **Content Validity - Box D** |  |  |  |  |  |  |  |  |  |  |
| --- | --- | --- | --- | --- | --- | --- | --- | --- | --- | --- |
| PCO | Condition | Original Article | Reference Article | 1 | 2 | 3 | 4 | 5 | Overall Score |  |
| VAS | Cuboid | Jennings 2005 | Salo | Excellent | Excellent | Good | Excellent | Excellent | Good | **GOOD** |
| Numeric Pain Rating Scale (NPRS) | LBP | Walston | La Montagne | Poor | Excellent | Good | Poor | Poor | Poor | **POOR** |
| FABQ | LBP | Walston | Wilson | Excellent | Excellent | Good | Excellent | Fair | Fair | **FAIR** |

| **Structural Validity - Box E** |  |  |  |  |  |  |  |  |  |  |  |
| --- | --- | --- | --- | --- | --- | --- | --- | --- | --- | --- | --- |
| PCO | Condition | Original Article | Reference Article | 1 | 2 | 3 | 4 | 5 | 6 | 7 | Overall Score |
| VAS | Cuboid Syndrome | Jennings 2005 | Crossley | Yes | Good | Fair | Fair | Poor | Poor | N/A | **POOR** |
| FABQ | LBP | Walston | Wilson | Yes | Good | Fair | Fair | Fair | Poor | N/A | **POOR** |
| CAS (Constipation Assessment scale) | Constipation and cerebral palsy | Tarsuslu | Woolery | Yes | Good | Fair | Fair | Fair | Poor | N/A | **POOR** |
| VAS | Constipation and cerebral palsy | Tarsuslu | Scott | Yes | Good | Fair | Excellent | Poor | Poor | N/A | **POOR** |

| **Criterion Validity - Box H** |  |  |  |  |  |  |  |  |  |  |  |
| --- | --- | --- | --- | --- | --- | --- | --- | --- | --- | --- | --- |
| PCO | Condition | Original Article | Reference Article | 1 | 2 | 3 | 4 | 5 | 6 | 7 | Overall Score |
| Crying time from a 24h crying diary | Infantile Colic | Miller | St. James | Good | Fair | Fair | Good | Fair | Poor | N/A | **POOR** |
| Crying time from a 24h crying diary | Infantile Colic | Miller | Barr | Good | Fair | Poor | Good | Fair | Excellent | N/A | **POOR** |
| Crying time diary | Infantile Colic | Browning | Barr | Good | Fair | Poor | Good | Fair | Excellent | N/A | **POOR** |
| Sleep diary (as part of crying diary data obtained) | Infantile Colic | Browning | Kirjavian | Excellent | Fair | Fair | Good | Fair | Excellent | N/A | **FAIR** |

| **Responsiveness** | | **- Box I** |  |  |  |  |  |  |  |  |  |  |  |  |  |  |  |  |  |  |
| --- | --- | --- | --- | --- | --- | --- | --- | --- | --- | --- | --- | --- | --- | --- | --- | --- | --- | --- | --- | --- |
| PCO | Condition | | Original Article | Reference Article | 1 | 2 | 3 | 4 | 5 | 6 | 7 | 8 | 9 | 10 | 11 | 12 | 13 | 14 | 15 | Overall Score |
| VAS | Cuboid Syndrome | | Jennings | Crossley | Good | Fair | Poor | Excellent | Excellent | Excellent | Fair | Poor | Good | Good | Excellent | Good | Poor | Excellent | N/A | **POOR** |
